# Determination of Intestinal Viral Loads and Distribution of Bovine Viral Diarrhea Virus, Classical Swine Fever Virus, and Peste Des Petits Ruminants Virus: A Pilot Study

**DOI:** 10.3390/pathogens10091188

**Published:** 2021-09-14

**Authors:** Tinka Jelsma, Joris J. Wijnker, Bregtje Smid, Eline Verheij, Wim H. M. van der Poel, Henk J. Wisselink

**Affiliations:** 1 Department of Virology, Wageningen Bioveterinary Research (WBVR), Wageningen University & Research (WUR), P.O. Box 65, 8200 AB Lelystad, The Netherlands; bregtje.smid@wur.nl (B.S.); Eline.Verheij@wur.nl (E.V.); wim.vanderpoel@wur.nl (W.H.M.v.d.P.); 2 Department of Population Health Sciences, Institute for Risk Assessment Sciences, Faculty of Veterinary Medicine, Utrecht University, P.O. Box 80.175, 3508 TD Utrecht, The Netherlands; j.j.wijnker@uu.nl; 3 Department of Infection Biology, Wageningen Bioveterinary Research (WBVR), Wageningen University & Research (WUR), P.O. Box 65, 8200 AB Lelystad, The Netherlands; henk.wisselink@wur.nl

**Keywords:** bovine viral diarrhea virus, classical swine fever virus, peste des petits ruminants virus, viral load, intestine, mucosa, submucosa, muscular layer/serosa, natural casing

## Abstract

The aim of this pilot study was to determine viral loads and distribution over the total length, at short distances, and in the separate layers of the intestine of virus-infected animals for future inactivation studies. Two calves, two pigs, and two goats were infected with bovine viral diarrhoea virus (BVDV), classical swine fever virus (CSFV), and peste des petits ruminants virus (PPRV), respectively. Homogenously distributed maximum BVDV viral loads were detected in the ileum of both calves, with a mean titer of 6.0 log_10_ TCID_50_-eq/g. The viral loads in colon and caecum were not distributed homogenously. In one pig, evenly distributed CSFV mean viral loads of 4.5 and 4.2 log_10_ TCID_50_-eq/g were found in the small and large intestines, respectively. Mucosa, submucosa, and muscular layer/serosa showed mean viral loads of 5.3, 3.4, and 4.0 log_10_ TCID_50_-eq/g, respectively. Homogenous distribution of PPRV was shown in the ileum of both goats, with a mean viral load of 4.6 log_10_ TCID_50_-eq/g. Mean mucosa, submucosa, and muscular layer/serosa viral loads were 3.5, 2.8, and 1.7 log_10_ TCID_50_-eq/g, respectively. This pilot study provides essential data for setting up inactivation experiments with intestines derived from experimentally infected animals, in which the level and the homogeneous distribution of intestinal viral loads are required.

## 1. Introduction

Natural casings are edible sausage containers destined for human consumption, and the majority of these products consist of the submucosa layer of porcine, sheep, and small ruminant’s intestines. For cattle casings, the intact intestine, instead of the submucosa layer, is used. After slaughter, the intestines are removed from the animal, processed, and preserved by a salt treatment for 30 days. Natural casings are sourced and traded worldwide, which is associated with possible risks of contamination when casings are produced from animals living in areas where viral diseases are prevalent. Previous in vivo studies on the efficacy of the standard operating procedures for the production of casings in inactivating foot and mouth disease virus (FMDV) resulted in non-sufficient viral titers to measure virus inactivation, which is an important limitation when performing inactivation studies by animal experiments [1,2]. To circumvent this limitation, an in vitro 3D collagen matrix model was developed to determine the inactivation kinetics of four contagious animal viruses by either saturated sodium chloride (NaCl) or phosphate-supplemented NaCl (P-salt) at 4 °C, 12 °C, 20 °C, and 25 °C [3]. Because of the 3D collagen model usability, the European Food Safety Authority (EFSA) Panel on Animal Health and Welfare (AHAW) strongly recommends validation of this model by actual animal studies [4]. This resulted in a research strategy ultimately leading to an in vivo validation experiment [5]. This strategy started with a systematic review (SR), searching for published intestinal viral loads of 14 animal viruses to identify viruses with a high intestinal viral load. The SR showed that information about viral loads in the intestines was very limited and that major gaps concerning the location and distribution of the virus in the intestines exist. Additionally, the SR included a meta-analysis about published inactivation data of three viruses, i.e., bovine viral diarrhea virus (BVDV), classical swine fever virus (CSFV), and peste des petits ruminants virus (PPRV), which were selected based on their high intestinal viral loads [6]. The aim was to select one of these viruses for a final in vivo validation (inactivation) experiment using the intestines derived from virus-infected animals. However, before enrolling this validation study, four basic research questions had to be addressed: (i) Will the proposed animal infection models result in sufficient intestinal viral loads to measure at least 2 log_10_ reduction during the validation experiment? (ii) How is the virus distributed over the total length of the intestines? (iii) How is the virus distributed over the different layers of the intestines (mucosa, submucosa, muscular layer/serosa)? (iv) Is there a homogeneous distribution of the virus, measuring less than 1 log_10_ variation, over short distances within the intestines? Studies determining viral loads and virus distribution in a methodological manner throughout the entire intestinal tract of infected animals were not described before [6]. The answers to these questions are crucial for a proper sampling over a large area (~100 samples/1.5 m intestine) requiring a homogenous distribution of viral loads to prevent inconclusive results due to the lack of homogeneity. This pilot study was performed with three viruses, BVDV, CSFV, and PPRV, because of their previously described high viral loads.

## 2. Results

### 2.1. Animal Studies

#### 2.1.1. BVDV

**Figure 1:**
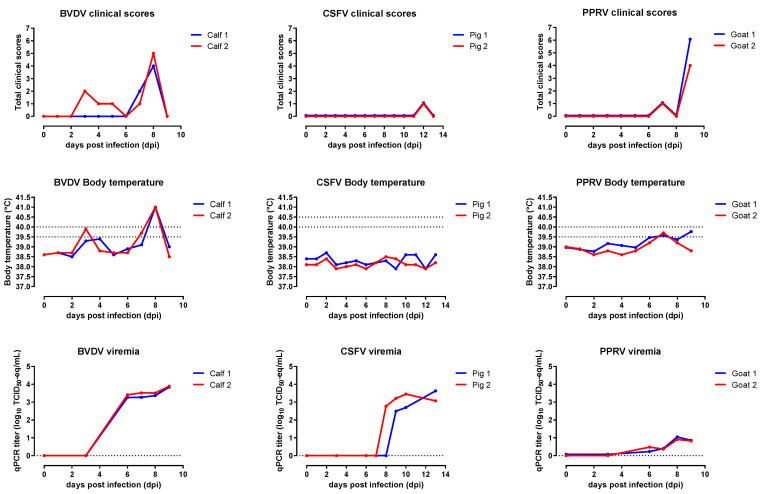
Clinical scores, body temperatures, and viremia results. Dotted lines at body temperatures indicate the temperature range regarding the onset of fever. Dotted line at viremia indicate the detection limit of the qPCR.

Both calves were active, appeared healthy, and were free of pestivirus antibodies at the start of the study. The clinical signs observed consisted mainly of an increased respiratory rate and fever. Calf 1 showed mild clinical signs between 2 and 6 days post-infection (dpi), calf 2 showed no clinical signs during that period, but nasal discharge was observed at 8 dpi. From 0 dpi, the body temperature of both calves started at 38.6 °C and showed a biphasic course typical of a BVDV infection. The first temperature peak was measured at 3–4 dpi, reaching 39.4 °C and 39.9 °C for calf 1 and 2, respectively. One day later, body temperatures normalized until a second peak reaching 41 °C, in both calves, at 8 dpi. Viremia started from 3 dpi, and similar qPCR titers for both calves were observed, reaching a maximum titer of approximately 3.9 log_10_ TCID_50_-eq/mL at 9 dpi. On that day, the animals were euthanized, and normal body temperature and no clinical signs were observed (Figure 1).

#### 2.1.2. CSFV

Both pigs were active, appeared healthy, and were free of pestivirus antibodies at the start of the study. During the entire study, no fever was observed, and at 12 dpi, the pigs showed some loss of appetite. Viremia started 8 and 9 dpi for pig 2 and 1, respectively. The maximum blood titer equivalent (3.5 log_10_ TCID_50_-eq/mL) in pig 2 was reached 10 dpi. The blood titers of pig 1 were still increasing to 3.6 log_10_ TCID_50_-eq/mL at 13 dpi and on that day, the pig was euthanized (Figure 1).

#### 2.1.3. PPRV

Both goats were active and healthy at the start of the study. At 7 dpi, both goats had a body temperature higher than 39.5 °C but less than 40 °C, resulting in a score of one. Their behavior was normal throughout the study; however, at 9 dpi, nasal discharge was observed in both animals. On this day (the end of the study), mucosal lesions in the mouth were observed in both goats, with scores of 1 and 2 for goat 2 and 1, respectively. During this study, elevated body temperatures were measured, rising from 39.0 °C/38.9 °C to 39.7 °C on 7 and 9 dpi for goats 2 and 1, respectively. At the end of the study (9 dpi), goat 2 had a normal body temperature, while goat 1 still had fever (39.7 °C). Viremia in both goats developed very similarly, and the first positive qPCR results were obtained 6 dpi. At 8 dpi, the maximum PPRV titer equivalents were 1.8 and 1.7 log_10_ TCID_50_-eq/mL for goats 1 and 2, respectively (Figure 1).

### 2.2. Intestinal Viral Loads

#### 2.2.1. BVDV

**Figure 2:**
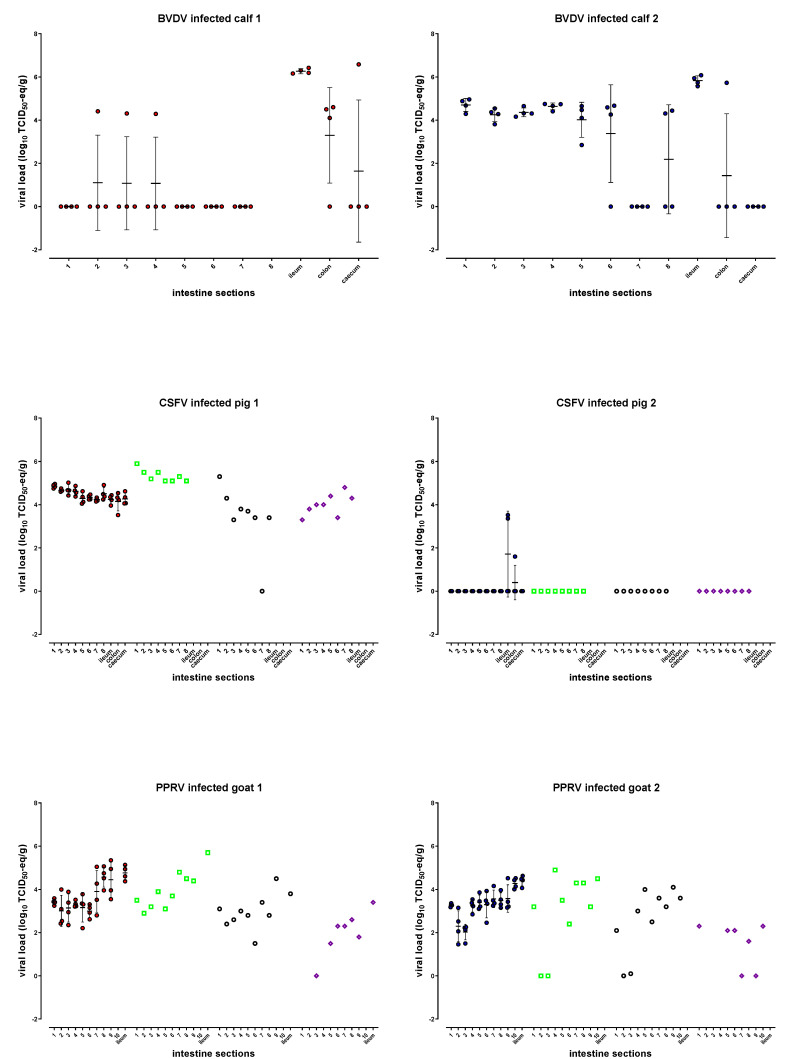
Mean and individual intestinal viral loads per virus expressed as log_10_ TCID_50_-eq/g are indicated by the red (animal 1) and blue circles (animal 2), error bars indicate the standard deviation. The viral loads in the different intestinal layers, mucosa (green □), submucosa (black ○), and muscular layer/ serosa (purple ◊) are based on a single sample per intestine section. For raw data, see Table S1 (BVDV), Table S2 (CSFV) and Table S3 (PPRV).

As indicated in Figure 2 and Table S1, sufficient maximum mean BVDV viral loads of 6.3 and 5.8 log_10_ TCID_50_-eq/g were found in the ileum of both calves, and the amount of virus was homogenously distributed over short distances, showing ≤0.5 log_10_ variation (SD 0.1–0.2). A homogenous distribution was also found in the proximal sections 1–4 of the small intestines of calf 2, including duodenum and jejunum, but with lower levels of BVDV (~4.5 log_10_ TCID_50_-eq/g). The viral loads in the other sections, including colon and caecum, were not homogeneously distributed (Figure 2 and Table S1). In calf 1, most sections besides the ileum were negative, and only single samples were BVDV-positive in sections 2–4, colon, and caecum. All cleaning control samples were negative.

To summarize, the optimal location for future BVDV sampling is in the ileum because of sufficient viral loads that were evenly distributed in both calves.

#### 2.2.2. CSFV

All intestine samples of pig 1 were positive, resulting in sufficient mean titers ranging from 4.2 (section 7 and colon) to 4.9 (section 1) log_10_ TCID_50_-eq/g. The samples per section showed homogenous distribution within 1 log_10_ variation (SD 0.06–0.4). The titer of the mucosa of pig 1 ranged from 5.1 (section 6) to 5.9 (section 1) log_10_ TCID50-eq/g, that for the submucosa ranged from negative (section 7) to 5.3 (section 1) log_10_ TCID_50_-eq/g, and the muscular layer/serosa values ranged from 3.3 (section 1) to 4.8 (section 7) log_10_ TCID_50_-eq/g (Figure 2 and Table S2). A paired t test analysis showed that the mucosa titers significantly differed from the submucosa (*p* 0.0068) and muscular layer/serosa (*p* 0.0008) titers, while no significant differences were found between the submucosa and muscular layer/serosa.

The average titer of all small intestine samples (sections 1–8) was 4.5 log_10_ (SD 0.1), while the mean submucosa titer was 3.4 log_10_ (SD 1.5), and only the submucosa titer in section 1 was higher than the titers of all four whole intestine samples from that section. No layer samples were taken from the ileum. None of the intestine samples of pig 2 were positive, except for two samples in the ileum and one caecum sample; none of the separate layers were positive (Figure 2 and Table S2). All cleaning control samples were negative.

To summarize, CSFV sampling can be performed in all sections of the intestinal tract, resulting in sufficient viral loads which are homogenously distributed. The viral loads in the submucosa layers of pig 1 were in most sections lower than in the whole intestine samples. However, this was observed only in one out of two animals, and most samples of the other pig were negative.

#### 2.2.3. PPRV

PPRV was homogenously distributed in the ileum of both goats within 1 log_10_ variation (SD 0.3/0.2), resulting in sufficient maximum mean viral loads of 4.8 (goat 1) and 4.4 (goat 2) log_10_ TCID_50_-eq/g. The samples of all other intestine sections were positive, showing mean titers ranging from 3.0 (section 2) to 4.6 (section 8) and 2.0 (section 3) to 4.3 (section 10) log_10_ TCID_50_-eq/g for goats 1 and 2, respectively (Figure 2 and Table S3). Sections 1, 4, 6 (goat 1) and 1, 3–5, 7, 8, 10 (goat 2) showed less than 1 log_10_ variation, and the mean titers gradually increased with approximately 1 log_10_ from section 7 towards the ileum (~8 meter). The viral load in the separate layers of the small intestine, mucosa, submucosa, and muscular layer/serosa were determined, and sections 1, 2, and 4 of goat 1 and sections 2, 3, and 4 of goat 2 resulted in a mixture of mucosa and muscular layer/serosa due to technical difficulties while learning the scraping technique to separate the different layers; the related results are indicated with an asterisk in Table S3 and, therefore, may not reflect the actual titers. When excluding these sections for the mucosa and muscular layer/serosa, the titers of the mucosa of goat 1 ranged from 3.1 (section 5) to 5.7 (ileum) log_10_ TCID_50_-eq/g, those of the submucosa ranged from 1.5 (section 6) to 4.5 (section 9) log_10_ TCID_50_-eq/g, and those of the muscular layer/serosa ranged from negative (section 3) to 3.4 (ileum) log_10_ TCID_50_-eq/g. The mucosa titers of goat 1 significantly differed from the submucosa (*p* 0.0031) and muscular layer/serosa (*p* < 0.0001) titers, while no significant differences were found between submucosa and muscular layer/serosa.

For goat 2, the mucosa titers ranged from 2.4 (section 6) to 4.5 (section 10) log_10_ TCID_50_-eq/g, those of the submucosa ranged from negative (section 2) to 4.1 (section 9) log_10_ TCID_50_-eq/g, and those of the muscular layer/serosa ranged from negative (sections 7 and 9) to 2.3 (section 1 and 10) log_10_ TCID_50_-eq/g. The muscular layer/serosa titers of goat 2 significantly differed from the mucosa (*p* 0.0065) and submucosa (*p* 0.0223) titers, while no significant differences were found between the submucosa and the mucosa. During homogenization of the frozen intestine samples of goat 1, seven cleaning controls were performed, of which two were positive (2.2 and 1.0 log_10_ TCID_50_-eq/g), and the others were negative. Five cleaning controls were performed for goat 2, of which one was found positive (1.3 log_10_ TCID_50_-eq/g), and the others were negative. Because the majority of the cleaning controls were negative and most intestine samples showed higher titers, the influence of contamination on the final intestine results was estimated to be relatively low.

To summarize, sampling PPRV in the ileum will result in sufficient maximum titers and minimal variation over short distances. The majority of the submucosa viral loads were lower than those of whole intestine samples.

## 3. Discussion

A prerequisite for testing intestinal virus inactivation is a homogeneous distribution of sufficient viral loads throughout the entire intestine. No previous studies on this subject have been performed [6]. The present study fills this gap by answering the four research questions indicated in the introduction. Sufficient viral loads of BVDV, CSFV, and PPRV were found in the small intestines of their host animals, making it possible to measure at least a 2 log_10_ reduction (99% reduction) in a future inactivation experiment (question (i)). The viral loads were evenly distributed either over the total length (CSFV) or in the ileum (BVDV and PPRV) of the small intestines, showing minimal variation (≤1 log_10_) in virus titers over short distances, which is important to measure the effect of inactivation (questions (ii) and (iv)). Regarding the different layers, CSFV and PPRV showed maximum and minimal viral loads in the mucosa and muscular layer/serosa layers, respectively (question (iii)). Specific to their purpose and relevant to international trade, the viral loads found in the submucosa layer (natural casing) were in general lower than in the whole intestine samples and showed more variation between the different sections. Therefore, for any future inactivation experiments, whole intestine sampling is preferred over sampling only the submucosa layer, and represents a worst-case scenario because of its higher viral loads.

Previous intestinal BVDV viral loads from 4 up to 7.3 log_10_ TCID_50_/g in in the ileum during a period of 6 to 10 dpi [7,8,9] confirmed the findings of the present study.

The CSFV intestinal viral loads found in the present study are in line with those of previous studies, showing maximum titers of 6.8 and 4.7 log_10_ TCID_50_/g in the ileum and jejunum, respectively [5,10]. Moreover, the inactivation study following this pilot analysis confirmed the homogeneous distribution of viral loads [5]. From outbreak situations and experimental data, it is known that CSFV infection of older pigs (in the present study at slaughter age of 6 months) may result in subclinical signs [11,12,13,14,15]. The clinical results obtained here were confirmed in the inactivation study, showing no fever and mild clinical signs [5]. Thus far, the presence of CSFV was not quantified per intestinal layer, mucosa, submucosa, and muscular layer/serosa in infected pigs. However, the presence of CSFV was detected by immunohistochemistry (IHC) in the superficial and crypt mucosal epithelial cells of the ileum and in macrophages and lymphocytes of the Peyer’s patches [16,17]. Previous studies involving natural casings derived from CSFV-infected pigs have shown the presence of infectious viruses in natural casings (submucosa) after processing; however, in none of these studies, the amount of the virus was quantified [18,19,20].

A recent study showed maximum viral loads of a local Bangladeshi PPRV strain BD/PPRV/2015/1 by qPCR in the duodenum, jejunum, and ileum, the latter showing the highest amount of virus, similar to the results of the present study [21]. During the past years, IHC studies have shown the presence of numerous PPRV strains in the small intestines, confirming the involvement of the mucosal layer (lamina propria, surface, and crypt epithelial cells) as well as of the submucosa layer (Peyer’s patches) during infection [22,23,24,25,26,27,28].

The present study shows the limitations of animal testing, which resulted in negative or very low intestinal viral loads in one of the two BVDV- and CSFV-infected animals, despite the fact that these viruses were selected because of their high viral loads in the intestines and the documented application of similar inoculation doses and routes [6,9,10,28]. Increasing the power of the experiment was not in line with the 3R’s disciplines of replacement, reduction, and refinement regarding animal welfare, because this was an exploratory experiment. The urge for replacement of animal testing was the primary incentive for the development of the 3D collagen matrix model [3]. The current study confirms this need, allowing testing the inactivation of sufficient and homogenous levels of virus without the use of animals and without the need to compromise between whole intestine or submucosa.

The present study focused on experimentally infected animals during an acute infection, but in a field situation, BVDV- and PPRV-persistently infected (PI) animals may also exist, shedding the virus for a long period of time [29,30]. Although PI animals were not the subject of this study, it would be quite interesting to investigate in future studies whether the intestinal viral titers of PI animals contain comparable levels of viral loads as those in acutely infected animals.

In conclusion, the results of this pilot study show that it is possible to set up an inactivation experiment with the intestines of animals experimentally infected with one of the examined viruses. To measure inactivation with sufficient viral loads, the whole intestine should be used instead of the submucosa layer (natural casing). Furthermore, these animal experiments should be executed with great attention to timing to determine the right moment for necropsy.

## 4. Materials and Methods

### 4.1. Viruses

In this study, three virulent virus strains were used: CSFV strain Paderborn 277, isolated in 1997 during the classical swine fever outbreak in the Paderborn area of Germany [31], BVDV type 1b strain 4800 (non-cytopathic), isolated from a persistently infected calf in 1993 [32], and PPRV strain CI/89, a pathogenic field strain isolated in Ivory Coast in 1989 and kindly provided by the Pirbright Institute (UK) [33].

### 4.2. Animal Studies

The animal experiment was approved by the national ethical committee (CCD) under code AVD 401002016687. Animals (pigs, calves, goats), at the age of approximately 6 months, were obtained from conventional farms with a high health status in the Netherlands. Before arrival at the high containment unit (HCU) facilities of Wageningen Bioveterinary Research (WBVR), the selected pigs and calves were free of pestivirus antibodies, as determined using the pan-pesti NS3 ELISA (Priocheck BVDV Ab, Thermofisher Scientific, Breda, The Netherlands). All animals were inoculated via the intranasal route; for CSFV and BVDV, these is a common route for experimental infection, while for PPRV, either the subcutaneous or the intranasal route are commonly applied. Several PPRV studies showed no differences in the final outcome depending on the infection route; therefore, the intranasal route was chosen, since this route reflects the natural way of PPRV transmission [33,34].

Two calves were infected with 2 mL 5 log_10_ TCID_50_/mL of BVDV (1 mL/nostril); the inoculation dose and route was based on a previous publication on the same strain of BVDV [9]. Two pigs were inoculated with 2 mL 5.0 log_10_ TCID_50_/mL CSFV (1 mL/nostril), based on previous publications [10,35]. Two goats were inoculated with 1 mL 4.8 log_10_ TCID_50_/mL PPRV (0.5 mL/nostril), which is similar the doses employed in previous publications on this strain [33,36]. The health of the animals was monitored daily by rectal temperature measurements, and clinical observations were based on previously described clinical scoring lists for each virus [24,37,38]. Each individual clinical score added up to a total score per day to monitor whether humane endpoints (HEP) were reached. EDTA-stabilized blood samples were taken on day 0 (prior to inoculation), day 3, and daily from day 6 till the end of the study and analyzed immediately by quantitative real-time PCR (qPCR) to determine the relative virus titer after 1:1 dilution in PBS. All animals were euthanized in the acute phase of the disease, aiming at the peak of viremia.

### 4.3. Intestine Sampling

The viral load in the intestines of experimentally infected calves, pigs, and goats was determined over the full-length of the small and large intestines. Additionally, the viral loads were determined in the separate layers of the small intestines, i.e., mucosa, submucosa, muscular layer/serosa (Table S4). Feces were manually squeezed out of the small intestines and flushed with water from the large intestines. Based on its length, the small intestines were divided in either 8–9 (calves/pigs) or 10 equal sections (goats). The length of each section varied from 3.2 to 3.6 m for calves and 1.9 to 2.1 m for pigs, and was 1.7 m for goats. From the center of each section, four consecutive samples with a length of 1 to 4 cm were taken, and each cut was made with a clean disposable knight to prevent cross-contamination. Each sample was weighed and weighed between 1 and 7 g. Because the ileum was located at the end of section 8 (pigs/calves) or 10 (goats), either additional sampling in the ileum was performed, which was the case for calf 2, both pigs, and goat 2, or the center sampling of the last section was replaced by sampling in the ileum of calf 1 and goat 1 (Table S4). From the small intestines of the PPRV-infected goats and CSFV-infected pig mucosa, submucosa and muscular layer/serosa samples were taken of each section (~20 cm) next to the four other samples. To approach a situation similar to the production process, the separation of the different layers was performed manually by an expert from the casing industry with the use of a specific scraping device. Most separations were performed by the expert, who also provided training, transferring his skills, for future studies. Briefly, after manually removing the feces, each small intestine section was cleaned by a 20 min soak in clean warm water (47 °C), followed by rinsing at the water tap. Subsequently, the mucosa was removed and collected in a first round with the scraping device, followed by a firmer scraping, removing and collecting the muscular layer/serosa, which finally resulted in the production and collection of the submucosa layer. The separate layers were verified macroscopically; however, some small residual amount of either the mucosa or the muscular layer or both might be present in the submucosa, as was previously described in histological studies of manually and mechanically processed sheep casings [39]. The cleaning process of porcine casings was quite similar to that of the sheep casings; thus, the conclusions obtained for sheep casings are also valid for porcine casings [40]. The intestines of the calves were processed differently, because, in this case, only the whole intestine is used as a sausage casing; therefore, it was not relevant to determine the viral load in the different layers [41]. From the large intestines of CSFV-infected pigs and BVDV-infected calves, 4 consecutive samples were taken from the caecum and at the center of the colon. Each sample was weighed and weighed between 2 and 7 g. The total lengths of the large intestine of pigs 1 and 2 were 4.9 m and 3.4 m, respectively. The total lengths of the large intestines of calf 1 and 2 were measured but not noted. Colon and caecum were not sampled from PPRV-infected goats because these intestinal parts have very little relevance for the casing industry, as indicated in Annex 1 on the ENSCA website [42].

### 4.4. Intestinal Viral Loads

The intestinal samples were immediately stored at −70 ± 5 °C; thereafter, the frozen intestinal samples were ground with the use of a cryogenic tissue lyser (Proficook), as previously described [5]. To obtain 20% tissue homogenates, cold medium, in a quantity of 4 volumes of the weight of each sample was added, e.g., 12 mL of medium to 3 g of sample. After processing each sample, the tissue lyser was decontaminated with Decon 90 (Agar Scientific, Stansted, UK), followed by rinsing with medium to prevent cross-contamination; random cleaning control samples, after medium rinsing, were taken. BVDV samples were homogenized in the presence of Eagle’s Minimum Essential Medium (EMEM), 2% fetal bovine serum (FBS), and 5% antibiotics; CSFV samples in EMEM, 5% FBS, and 10% antibiotics; PPRV samples in Dulbecco’s Modified Eagle’s Medium (DMEM), 10% FBS, 1% l-glutamine (2 mM), 100 U/mL penicillin, and 100 µg/mL streptomycin. All homogenates and cleaning control samples were clarified by centrifuging at 1966–2570× *g* for 10 min at 4 °C and stored at −70 °C until qPCR testing.

### 4.5. Quantitative RT-PCR

To determine the viral load and homogeneity of the virus distribution over each intestine section, all samples were tested by quantitative RT-PCR, detecting BVDV, CSFV, or PPRV [43,44,45,46]. To quantify the amount of virus DNA/RNA present, a standard curve was obtained using known titers of BVDV 4800, CSFV Paderborn, or PPRV CI/89, in parallel the analysis of the homogenized samples; therefore, the results are expressed in log_10_ TCID_50_ equivalents (TCID_50_-eq). Because PCR detects viral RNA derived from infectious and non-infectious virus, the qPCR results represent a relative measure for the viral load. In the validation experiment following the present pilot study, it was shown that the equivalent qPCR titers were similar to the virus titers obtained by virus titration in cultured cells [5]. 

## Data Availability

All data presented in this study are available in this manuscript and supplemental materials.

## Supplementary Materials

The following are available online at https://www.mdpi.com/article/10.3390/pathogens10091188/s1, Table S1: BVDV qPCR results in small and large intestine sections; viral loads are expressed as log_10_ TCID_50_-eq/g, Table S2: CSFV qPCR results in small and large intestines; viral loads are expressed as log_10_ TCID_50_-eq/g, Table S3: PPRV qPCR results in small intestines; viral loads are expressed as log_10_ TCID_50_-eq, Table S4: Intestinal sampling scheme of the BVDV-infected calves, CSFV-infected pigs, and PPRV-infected goats.

## References

[1] Wijnker J.J., Haas B., Berends B.R. (2007). Removal of foot-and-mouth disease virus infectivity in salted natural casings by minor adaptation of standardized industrial procedures. Int. J. Food Microbiol..

[2] Wijnker J.J., Haas B., Berends B.R. (2012). Inactivation of foot-and-mouth disease virus in various bovine tissues used for the production of natural sausage casings. Int. J. Food Microbiol..

[3] Wieringa-Jelsma T., Wijnker J.J., Zijlstra-Willems E.M., Dekker A., Stockhofe-Zurwieden N., Maas R., Wisselink H.J. (2011). Virus inactivation by salt (NaCl) and phosphate supplemented salt in a 3D collagen matrix model for natural sausage casings. Int. J. Food Microbiol..

[4] EFSA (2012). Scientific opinion on animal health risk mitigation treatments as regards imports of animal casings. EFSA J..

[5] Jelsma T., Wijnker J.J., Smid B., Verheij E., Poel W.H.M.v.d., Wisselink H.J. (2019). Salt inactivation of classical swine fever virus and African swine fever virus in porcine intestines confirms the existing in vitro casings model. Vet. Microbiol..

[6] Jelsma T., Wijnker J.J., van der Poel W.H.M., Wisselink H.J. (2021). Intestinal Viral Loads and Inactivation Kinetics of Livestock Viruses Relevant for Natural Casing Production: A Systematic Review and Meta-Analysis. Pathogens.

[7] Pedrera M., Gómez-Villamandos J.C., Molina V., Risalde M.A., Rodríguez-Sánchez B., Sánchez-Cordón P.J. (2012). Quantification and determination of spread mechanisms of bovine viral diarrhoea virus in blood and tissues from colostrum-deprived calves during an experimental acute infection induced by a non-cytopathic genotype 1 strain. Transbound. Emerg. Dis..

[8] Marshall D.J., Moxley R.A., Kelling C.L. (1996). Distribution of Virus and Viral Antigen in Specific Pathogen-free Calves Following Inoculation with Noncytopathic Bovine Viral Diarrhea Virus. Vet. Pathol..

[9] Bruschke C.J.M., Weerdmeester K., Van Oirschot J.T., Van Rijn P.A. (1998). Distribution of bovine virus diarrhoea virus in tissues and white blood cells of cattle during acute infection. Vet. Microbiol..

[10] Wood L., Brockman S., Harkness J., Edwards S. (1988). Classical swine fever: Virulence and tissue distribution of a 1986 English isolate in pigs. Vet. Rec..

[11] Dahle J., Liess B. (1992). A review on classical swine fever infections in pigs: Epizootiology, clinical disease and pathology. Comp. Immunol. Microbiol. Infect. Dis..

[12] Blome S., Staubach C., Henke J., Carlson J., Beer M. (2017). Classical Swine Fever—An Updated Review. Viruses.

[13] (2015). CFSPH_CSFV. CSFV. https://www.cfsph.iastate.edu/Factsheets/pdfs/classical_swine_fever.pdf.

[14] Moennig V., Floegel-Niesmann G., Greiser-Wilke I. (2003). Clinical Signs and Epidemiology of Classical Swine Fever: A Review of New Knowledge. Vet. J..

[15] (2019). OIE_CSFV. https://www.oie.int/fileadmin/Home/eng/Health_standards/tahm/3.08.03_CSF.pdf.

[16] Narita M., Kawashima K., Kimura K., Mikami O., Shibahara T., Yamada S., Sakoda Y. (2000). Comparative Immunohistopathology in Pigs Infected with Highly Virulent or Less Virulent Strains of Hog Cholera Virus. Vet. Pathol..

[17] Izzati U.Z., Hoa N.T., Lan N.T., Diep N.V., Fuke N., Hirai T., Yamaguchi R. (2021). Pathology of the outbreak of subgenotype 2.5 classical swine fever virus in northern Vietnam. Vet. Med. Sci..

[18] McKercher P.D., Morgan D.O., McVicar J.W., Shuot N.J. Thermal processing to inactivate viruses in meat products. Proceedings of the 84th United States Animal Health Association.

[19] Helwig D.M., Keast J.C. (1966). Viability of virulent swine fever virus in cooked and uncooked ham and sausage casings. Aust. Vet. J..

[20] Wijnker J.J., Depner K.R., Berends B.R. (2008). Inactivation of classical swine fever virus in porcine casing preserved in salt. Int. J. Food Microbiol..

[21] Begum S., Nooruzzaman M., Islam M.R., Chowdhury E.H. (2021). A Sequential Study on the Pathology of Peste Des Petits Ruminants and Tissue Distribution of the Virus Following Experimental Infection of Black Bengal Goats. Front. Vet. Sci..

[22] Kul O., Kabakci N., Atmaca H.T., Ozkul A. (2007). Natural peste des petits ruminants virus infection: Novel pathologic findings resembling other morbillivirus infections. Vet. Pathol..

[23] Kumar P., Tripathi B.N., Sharma A.K., Kumar R., Sreenivasa B.P., Singh R.P., Dhar P., Bandyopadhyay S.K. (2004). Pathological and immunohistochemical study of experimental peste des petits ruminants virus infection in goats. J. Vet. Med. Ser. B Infect. Dis. Vet. Public Health.

[24] Pope R.A., Parida S., Bailey D., Brownlie J., Barrett T., Banyard A.C. (2013). Early Events following Experimental Infection with Peste-Des-Petits Ruminants Virus Suggest Immune Cell Targeting. PLoS ONE.

[25] Sahoo M., Dinesh M., Thakor J.C., Baloni S., Saxena S., Shrivastava S., Dhama K., Singh K., Singh R. (2020). Neuropathology mediated through caspase dependent extrinsic pathway in goat kids naturally infected with PPRV. Microb. Pathog..

[26] Toplu N. (2004). Characteristic and non-characteristic pathological findings in peste des petits ruminants (PPR) of sheep in the Ege district of Turkey. J. Comp. Pathol..

[27] Jagtap S.P., Rajak K.K., Garg U.K., Sen A., Bhanuprakash V., Sudhakar S.B., Balamurugan V., Patel A., Ahuja A., Singh R.K. (2012). Effect of immunosuppression on pathogenesis of peste des petits ruminants (PPR) virus infection in goats. Microb. Pathog..

[28] Truong T., Boshra H., Embury-Hyatt C., Nfon C., Gerdts V., Tikoo S., Babiuk L.A., Kara P., Chetty T., Mather A. (2014). Peste des petits ruminants virus tissue tropism and pathogenesis in sheep and goats following experimental infection. PLoS ONE.

[29] Schweizer M., Peterhans E. (2014). Pestiviruses. Ann. Rev. Anim. Biosci..

[30] Baron M.D., Diallo A., Lancelot R., Libeau G., Kielian M., Maramorosch K., Mettenleiter T.C. (2016). Chapter 1: Peste des Petits Ruminants Virus. Advances in Virus Research.

[31] Greiser-Wilke I., Zimmermann B., Fritzemeier J., Floegel G., Moennig V. (2000). Structure and presentation of a World Wide Web database of CSF virus isolates held at the EU Reference Laboratory. Vet. Microbiol..

[32] Bruschke C.J.M., Van Rijn P.A., Moormann R.J.M., Van Oirschot J.T. (1996). Antigenically different pestivirus strains induce congenital infection in sheep: A model for bovine virus diarrhea virus vaccine efficacy studies. Vet. Microbiol..

[33] Baron J., Bin-Tarif A., Herbert R., Frost L., Taylor G., Baron M.D. (2014). Early changes in cytokine expression in peste des petits ruminants disease. Vet. Res..

[34] El Harrak M., Touil N., Loutfi C., Hammouchi M., Parida S., Sebbar G., Chaffai N., Harif B., Messoudi N., Batten C. (2012). A reliable and reproducible experimental challenge model for peste des petits ruminants virus. J. Clin. Microbiol..

[35] Weesendorp E., Backer J., Stegeman A., Loeffen W. (2009). Effect of strain and inoculation dose of classical swine fever virus on within-pen transmission. Vet. Res..

[36] Herbert R., Baron J., Batten C., Baron M., Taylor G. (2014). Recombinant adenovirus expressing the haemagglutinin of peste des petits ruminants virus (PPRV) protects goats against challenge with pathogenic virus; a DIVA vaccine for PPR. Vet. Res..

[37] Strong R., La Rocca S.A., Paton D., Bensaude E., Sandvik T., Davis L., Turner J., Drew T., Raue R., Vangeel I. (2015). Viral Dose and Immunosuppression Modulate the Progression of Acute BVDV-1 Infection in Calves: Evidence of Long Term Persistence after Intra-Nasal Infection. PLoS ONE.

[38] Mittelholzer C., Moser C., Tratschin J.D., Hofmann M.A. (2000). Analysis of classical swine fever virus replication kinetics allows differentiation of highly virulent from avirulent strains. Vet. Microbiol..

[39] Koolmees P.A., Tersteeg M.H.G., Keizer G., Van Den Broek J., Bradley R. (2004). Comparative histological studies of mechanically versus manually processed sheep intestines used to make natural sausage casings. J. Food Prot..

[40] Koolmees P.A., Houben J.H. (1997). Inventory part: Histology and microbiology of hog and sheep casings. Colour Print Leaflet EU—CRAFT Project BRE 2. CT 94. 1495: Improved Treatment of Natural Sausage Casings for Quality Improvement in Automated Stuffing Processes.

[41] Wijnker J., Tersteeg M., Berends B., Vernooij J., Koolmees P. (2008). Quantitative Histological Analysis of Bovine Small Intestines before and after Processing into Natural Sausage Casings. J. Food Prot..

[42] ENSCA. http://www.ensca.eu/index.php?/eng/content/download/2936/22121/file/ENSCA%20cGGP%20revision%20VIII%2018-06-19.pdf.

[43] Hoffmann B., Depner K., Schirrmeier H., Beer M. (2006). A universal heterologous internal control system for duplex real-time RT-PCR assays used in a detection system for pestiviruses. J. Virol. Methods.

[44] Van Rijn P.A., Wellenberg G.J., Hakze-Van Der Honing R., Jacobs L., Moonen P.L.J.M., Feitsma H. (2004). Detection of economically important viruses in boar semen by quantitative RealTime PCR™ technology. J. Virol. Methods.

[45] Weesendorp E., Backer J., Loeffen W. (2014). Quantification of different classical swine fever virus transmission routes within a single compartment. Vet. Microbiol..

[46] van Rijn P.A., Boonstra J., van Gennip H.G.P. (2018). Recombinant Newcastle disease viruses with targets for PCR diagnostics for rinderpest and peste des petits ruminants. J. Virol. Methods.

